# Glyphosate and AMPA in Human Urine of HBM4EU Aligned Studies: Part A Children

**DOI:** 10.3390/toxics10080470

**Published:** 2022-08-12

**Authors:** Jurgen Buekers, Sylvie Remy, Jos Bessems, Eva Govarts, Loïc Rambaud, Margaux Riou, Janja Snoj Tratnik, Anja Stajnko, Andromachi Katsonouri, Konstantinos C. Makris, Annelies De Decker, Bert Morrens, Nina Vogel, Marike Kolossa-Gehring, Marta Esteban-López, Argelia Castaño, Helle Raun Andersen, Greet Schoeters

**Affiliations:** 1 Unit Health, VITO, Flemish Institute for Technological Research, 2400 Mol, Belgium; 2 Department of Environmental and Occupational Health, Santé Publique France, 94415 Saint-Maurice, France; 3 Department of Environmental Sciences, Jozef Stefan Institute, 1000 Ljubljana, Slovenia; 4 Cyprus State General Laboratory, Ministry of Health, Nicosia 2081, Cyprus; 5 Cyprus International Institute for Environmental and Public Health, Cyprus University of Technology, Limassol 3036, Cyprus; 6 PIH, Knowledge Center for Environment and Health, 2000 Antwerp, Belgium; 7 Department of Social Sciences, University of Antwerp, 2000 Antwerp, Belgium; 8 German Environment Agency (UBA), Berlin, 06844 Dessau-Roßlau, Germany; 9 Instituto de Salud Carlos III, National Centre for Environmental Health, 28220 Madrid, Spain; 10 Department of Public Health, University of Southern Denmark, 5000 Odense, Denmark; 11 Department of Biomedical Sciences, University of Antwerp, 2610 Antwerp, Belgium

**Keywords:** HBM4EU, glyphosate, AMPA, exposure, HBM, children

## Abstract

Few data are available on the exposure of children to glyphosate (Gly) in Europe. Within HBM4EU, new HBM exposure data were collected from aligned studies at five sampling sites distributed over Europe (studies: SLO CRP (SI); ORGANIKO (CY); GerES V-sub (DE); 3XG (BE); ESTEBAN (FR)). Median Gly concentrations in urine were below or around the detection limit (0.1 µg/L). The 95th percentiles ranged between 0.18 and 1.03 µg Gly/L. The ratio of AMPA (aminomethylphosphonic acid; main metabolite of Gly) to Gly at molar basis was on average 2.2 and the ratio decreased with higher Gly concentrations, suggesting that other sources of AMPA, independent of metabolism of Gly to AMPA in the monitored participants, may concurrently operate. Using reverse dosimetry and HBM exposure data from five European countries (east, west and south Europe) combined with the proposed ADI (acceptable daily intake) of EFSA for Gly of 0.1 mg/kg bw/day (based on histopathological findings in the salivary gland of rats) indicated no human health risks for Gly in the studied populations at the moment. However, the absence of a group ADI for Gly+AMPA and ongoing discussions on e.g., endocrine disrupting effects cast some uncertainty in relation to the current single substance ADI for Gly. The carcinogenic effects of Gly are still debated in the scientific community. These outcomes would influence the risk conclusions presented here. Finally, regression analyses did not find clear associations between urinary exposure biomarkers and analyzed potential exposure determinants. More information from questionnaires targeting exposure-related behavior just before the sampling is needed.

## 1. Introduction

Glyphosate, (Gly; C3H8NO5P; CAS RN 1071-83-6) often used as its glyphosate isopropylamine salt (C3H9N.C3H8NO5P; CAS RN 38641-94-0), is the active ingredient in broad-spectrum herbicide plant-protection products used worldwide, such as Roundup. The main degradation product of Gly in the environment and in humans is aminomethylphosphonic acid (AMPA; CAS RN 1066-51-9). It is considered that Gly and AMPA have a similar toxicological profile [1,2]. Different aspects lead to uncertainty about the safety assessment of Gly. On the exposure side, there is a lack of human biomonitoring (HBM) data, especially among the vulnerable groups, such as children, and the groups with potentially high exposure, such as those living in proximity to agricultural fields. For AMPA, there is uncertainty on the fraction originating from Gly degradation in the environment and contaminating the food chain which may contribute to AMPA internal exposure (direct exposure next to human internal metabolism of Gly to AMPA) [3]. Additionally, there are concerns that Gly in the environment could chelate essential and non-essential heavy metals, changing their bioavailability and, in consequence, human exposures to such metals [4]. On the effect side, there is a controversial debate about Gly carcinogenicity [5] and its endocrine disrupting properties [6], and concern on the recent findings on exposure-effect associations in epidemiological studies, including associations between Gly and preterm birth [7], gestational length [8,9] and anogenital distance [10]. Under REACH, the Risk Assessment Committee of ECHA again concluded, in its most recent scientific opinion (May 2022), that classifying glyphosate as a carcinogen is not justified [11], while in 2015, IARC concluded that Gly is probably carcinogenic (Group 2A) [5]. In a recent study of primary-school children in Cyprus, Makris et al. (2022) reported a significant association between urinary 8-OHdG (8-hydroxy-2′-deoxyguanosine), a DNA oxidative damage biomarker, and urinary AMPA [12]. What makes it more difficult is that some studies focused on Gly-based products such as Roundup, while others focused on the active ingredient itself [13]. The main sources of human exposure are assumed to be through the dietary intake of the residues in food and water and from inhalation of dust, or exposure in occupational settings or around crop fields. To assess the internal exposure, perform a proper risk assessment and unravel exposure determinants, quality-controlled HBM data are a prerequisite. This need was addressed by the HBM4EU project (www.hbm4eu.eu (accessed on 4 July 2022)), which collected high-quality biomonitoring data on internal human exposure in Europe to inform policy decisions [14].

## 2. Methods

### 2.1. Data

National or regional studies from five countries, which fulfilled predefined criteria, were aligned in the frame of HBM4EU, with the goal of collecting harmonized data on European children’s exposure to Gly and AMPA. The information about the involved studies was described in Gilles et al. (2021). The urinary data on Gly and AMPA were obtained from Germany (GerES V-sub; 2015–2017), Cyprus (ORGANIKO; 2017), Slovenia (SLO CRP; 2018), France (ESTEBAN; 2014–2016) and Belgium (3XG; 2019–2020). The sampling took place in different seasons. The participants were boys and girls of ages 6–11 years old, who provided urine samples in the period ranging from 2014–2020. The characteristics of the study participants are summarized in Table 1 and are described in more detail in Gilles et al. (2022), where additional information about the studies, including ethics, is presented [15,16].

The quality of the analytical measurements was controlled and assured by analyzing the urine samples in qualified laboratories, following a strict schedule [17]. All of the data were included for the analysis of the exposure determinants and risk assessment. The methodologies for handling data below the limits of detection (LOD) have been a long-recognized issue. The biomarker data are typically left-censored data; the data below a LOD for which the true value is unknown are often referred to as “left-censored”. The LOD and limit of quantification (LOQ) values were reported by the laboratories and applied to calculate detection frequencies. The values below the LOD/LOQ were not imputed for Gly and AMPA because of the low detection rates (below 50% in most of the studies).

The variables for assessing exposure differences were obtained through questionnaires from the specific studies. The questionnaires, collected within the new and ongoing HBM campaigns under HBM4EU, were harmonized. As some of the HBM campaigns were ongoing, research questions were harmonized post factum. Therefore, some of the information may be available in some of the questionnaires, while not in others. For the upcoming HBM studies, the base and substance-specific harmonized questionnaires are described in González-Alzaga et al. (2022) [18].

### 2.2. Statistics—Identifying Determinants of Exposure

The statistical analysis was performed in SPSS Statistics 28. Two types of analysis were performed. In the first individual country, the studies were separately analyzed, while in the second, the data from all of the countries were combined.

First analysis: The Gly and AMPA concentrations were dichotomized (0 for values below LOQ and 1 for values above). First, a logistic regression model was applied to identify the exposure determinants significantly explaining variability in exposure. The single variables (or determinants), which might explain the differences in Gly or AMPA concentrations, were tested with matrix (spot urine, first-morning urine), BMI and creatinine forced into the model. BMI was forced into the model because of the possible association with creatinine. Determinants considered were individual characteristics, dietary preferences, exposure-relevant behavior (e.g., use of pesticides by parents), sociodemographic information and sampling season (see Supplementary Material Tables S1 and S2 for detailed information). Second, a logistic multiple regression model was built including all of the variables with a *p* value ≤ 0.2 in the former analysis. By backward selection, starting with excluding the variable with the highest *p* value, only those variables with *p* ≤ 0.05 were kept in the analysis, including those forced into the model (matrix (spot or first-morning urine), BMI, creatinine). The reason why a larger *p* value was set as the threshold in the first analysis (*p* ≤ 0.2) compared to the second analysis (*p* ≤ 0.05) is that some of the associations became clearer when correcting for the co-variables. In order not to miss the co-variables, a less strict threshold was initially applied.

Second analysis: All of the data from all of the studies were combined and analyzed as a single dataset in a logistic regression model. Here, a value of 0.1 µg/L was uniformly applied as the LOQ cutoff for all of the studies. The data from the Belgian 3XG study were excluded from the analysis of AMPA, because the LOQ in this study was 0.2 µg/L and thus above the 0.1 µg/L level. The country was included in the model as a covariate next to BMI, matrix and creatinine. The determinants of variability were assessed one at a time (see Tables S1 and S2). No logistic multiple regression with backward selection was completed for all of the studies combined as not all of the determinants were available for all of the studies (see Tables S1 and S2).

### 2.3. Risk Assessment

The IARC classified Gly as probably carcinogenic (group 2A) [5], while ECHA-RAC again concluded that classifying Gly as carcinogen is not justified [11]. For the risk assessment, no HBM guidance value for exposure to Gly or AMPA yet exists. Based on the HBM data and information on kinetics, the external exposure values were estimated for Gly using reverse dosimetry [19]. The predicted daily intake (PDI) was compared with the proposed acceptable daily intake (ADI) of 0.1 mg/kg bw/day of EFSA [20].

A study of 12 human volunteers who ingested known levels of Gly and AMPA present in food (the quantity of AMPA was about 100-fold less than Gly), [21] showed that the urinary levels of Gly and AMPA differed approximately by only a factor of four. The authors concluded that, on average, 1% of the Gly dose was excreted in the urine (urinary excretion fraction = F_UE_ = 1%). The lowest observed F_UE_, in the study of Zoller et al. [21], was 0.57%.

The assessment of the risk from exposure to Gly was estimated using Equation (1). The concentration of glyphosate in the urine was multiplied by a standardized volume of urine per day and divided by a standardized body weight, multiplied by the urinary excretion fraction of glyphosate (assuming steady state) and multiplied by the proposed ADI established by EFSA (Equation (1). See also [22]). This calculation results in a predicted daily intake (PDI) of glyphosate versus the ADI (in percent):

(1) $$\%ADI=\frac{Gly_{conc}\times Vol_{urine}}{bw\times F_{UE}\times ADI}=\frac{PDI}{ADI}$$

where *Gly_conc_* is the concentration of glyphosate measured in urine; *Vol_urine_* is standardized as 0.7 L/day [23]; *bw* is bodyweight which is standardized at 30 kg; the *F_UE_* is set at 0.57% [21]; and *ADI* is the acceptable daily intake allowance for Gly (proposed at 0.1 mg/kg bw/day [20]; Point of Departure, PoD = NOAEL of 10 mg/kg/day based on histopathological findings in the salivary gland in a 2-year long rat study to which a standard assessment factor of 100 was applied [20]). At the moment, the ADI is equal to 0.5 mg/kg bw/day but it is proposed by EFSA to be reduced to 0.1 mg/kg bw/day.

## 3. Results and Discussion

### 3.1. Exposure

An overview of the Gly and AMPA urinary biomarkers measured in the HBM4EU-aligned studies is presented in Table 1 and Table 2. The HBM4EU result are discussed and compared with the published EU data (Table 3).

The HBM4EU data showed that children are internally exposed to Gly and AMPA across the EU. The median exposures were close to or below the LOQs (Table 1 and Table 2). The highest exposures to Gly and AMPA among the HBM4EU-aligned studies, and assured by the HBM4EU QA/QC program, were observed in Cyprus. A comparison of the HBM4EU-aligned studies with the published data from other European countries (Table 3) shows significantly higher median concentrations of Gly observed in Portugal (AM 1.77 µg/L) and Denmark (AM 1.96 µg/L). It should be noted that in both the Portuguese and Danish studies, the Gly was measured using ELISA [24,25], whereas in the HBM4EU-aligned studies the Gly was measured by GC/MS-MS or LC/MS-MS. It has been shown for other non-persistent chemicals with a short half-life, e.g., for bisphenol A, that ELISA lacks the sensitivity and specificity to measure exposure [28,29]. Overall, in the HBM4EU-aligned studies (Table 1 and Table 2), the P95 values varied between 0.18 and 1.03 µg/L for Gly and between 0.29 and 0.66 µg/L for AMPA, with the highest P95 values for both Gly (1.03) µg/L and AMPA (0.66 µg/L) observed in Cyprus.

These data create an overview of children’s exposure to Gly and AMPA within the EU. Children have a higher ingestion of food and drink per kilogram bodyweight compared to adults. They play outdoors and are more susceptible to pollutants, due to the fact that their organs are still developing [30]. HBM4EU substantially contributed to the provision of exposure data within the EU. Nevertheless, the exposure data on Gly and AMPA in children, especially among those living close to agricultural fields, remain limited, even though they are a vulnerable population.

### 3.2. AMPA to Glyphosate Ratio (AMPA/Gly)

**Figure 1:**
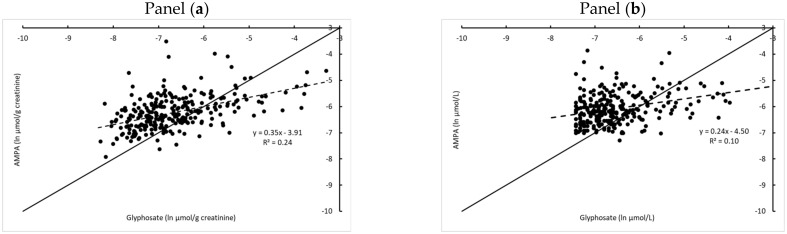
Pooled HBM4EU-aligned study data from five European countries (Belgium, Cyprus, Germany, Slovenia, France) for Gly and AMPA in children. Data are expressed on a natural logarithmic scale: panel (**a**) Creatinine-corrected concentrations (µmol/g creatinine); panel (**b**) Volume-based concentrations (µmol/L). The 1:1 line (full line) is indicated. Values below LOQ were excluded.

For each participant, the ratio of the AMPA to Gly was calculated and presented in Figure 1. Only the data from participants with quantifiable exposures (above the LOQ) for both Gly and AMPA (*n* = 263 data points) were included. From the creatinine-corrected concentrations (panel a), it could be derived that the AMPA/Gly ratio varied between 0.1 and 27.5 per study/participant with an average value of 2.2. In total, 194 of the 263 participants had an AMPA/Gly ratio higher than one. When regressing AMPA (in µmol/g creatinine) on the *Y*-axis against Gly (in µmol/g creatinine) on the *X*-axis, the slope of the linear fit is smaller than 1 (*p* < 0.001). The resulting trendline crossed the linear 1:1 line at 0.002 µmol Gly/g creatinine. The samples below that point (further to the left on the *X*-axis with the Gly concentrations) on average have higher AMPA than Gly concentrations. For the samples beyond that point, the Gly concentrations on average exceed the AMPA concentrations. In addition, based on the volumetric scale (panel b), the slope was <1.

This seems to suggest the existence of ‘autonomous’ or pre-exposure origins of AMPA (independent of the metabolism of Gly to AMPA in the monitored participants) i.e., external exposure to AMPA directly, as well as suggested earlier [3]. Gly-dependent AMPA is formed by metabolism in Gly-sprayed crops, as well as by microorganisms in the environment. AMPA has a long half-life in soil, which results in an accumulation in the environment [31]. In humans, AMPA is poorly metabolized [26,32]. In the human gut, a small fraction of Gly is metabolized into AMPA by the microflora, as observed in animal studies [33]. AMPA in the environment not only originates from Gly, but also from the massive use of amino-polyphosphonates [3]. These substances are used as detergents, fire retardants, anticorrosives and anti-scaling agents, and as complexing agents in the textile industry [34]. In addition, they are used as a membrane anti-fouling agent in water treatment. A similar moderate correlation between AMPA and Gly was also observed in published human studies from Sweden [35], Slovenia [27] and Germany [26], supporting the evidence of an additional intake of AMPA, next to the intake of Gly.

### 3.3. Risk Assessment (RA)

There is still a conflict of opinions between IARC and the EU bodies on the carcinogenicity of Gly. Genotoxicity in combination with carcinogenicity would mean there are no safe exposure levels. In 2015, IARC classified glyphosate as “probably carcinogenic to humans”, and concluded that there was “strong” evidence for genotoxicity. In contrast to IARC, the RAC committee confirmed their previous standpoint and concluded, in their scientific opinion that was published in May 2022, that the available scientific evidence did not meet the criteria to classify glyphosate for specific target organ toxicity, or as a carcinogenic, mutagenic or reprotoxic substance. The assumption taken regarding hazard identification might very well influence the hazard characterization, due to differing sensitivities to different endpoints.

The current data indicate widespread but relatively low Gly exposure (large part below LOD and LOQ) in children (Table 1). The quantified biomarkers allow an estimation of the internal exposure levels. By reverse dosimetry calculations, the daily intake can be predicted (PDI) and compared with the acceptable daily intake (ADI). Using urinary P95 values for Gly_conc_ (reasonable worst case), the PDI values were calculated and compared to the ADI (Table 4). The proposed ADI of 0.1 mg/kg bw/day is not exceeded for the European children who participated in the HBM4EU-aligned studies. However, in the general population living close to agricultural fields, the PDI could be closer to the ADI (see Section 3.4).

Importantly, AMPA has a similar toxicological profile as Gly [1,2], and this should be considered in the risk assessment. The combined exposure to both of the compounds should be considered, as was also suggested by the JMPR (the Joint FAO/WHO Meeting on Pesticide Residues), proposing a group-ADI for Gly+AMPA [36].

### 3.4. Exposure Determinants

To assess the determinants of exposure, the associations between urinary Gly and AMPA concentrations and data collected through questionnaires, were analyzed (See Supplementary Material Tables S1 and S2, for the first analysis).

The results from the logistic “multiple” regression model showed that for the Slovenian study, parental use of pesticides outdoors (garden, agriculture) resulted in higher concentrations of glyphosate in the children (Table 5). The use of herbicides containing glyphosate and applied near the home could result in higher urinary Gly. For the Belgian 3xG cohort, pets in the home and older age (age range limited to 6–8 y in 3XG) were associated with higher Gly concentrations, though the logistic regression did not show any significant associations between the more specific variables “having a cat or dog” and Gly urinary concentrations. It was speculated that more Gly was brought into the house by pets in the form of dust sticking to hair and paws, as was shown for other pesticides [37]. For the other studies, no significant associations were observed in the logistic multiple regression model at the 0.05 level.

BMI was forced in the model because of the possible association with creatinine. Without BMI in the model, the multiple regression model for SLO CRP would be significant at the 0.05 level (*p* = 0.037), and the variable use of pesticides outdoors was significant (OR = 2.39 (95%CI 1.08 to 5.30)). For the 3xG cohort, the model was still significant (*p* < 0.001) without BMI, and the variables of age (OR = 2.57 (95%CI 1.21 to 6.25)) and pets in the home (OR = 2.75 (95%CI 1.21 to 6.25)) were also significant.

The logistic multiple regression model did not show any significant associations with food items nor with the type of drinking water used. The number of participants using ground water for cooking or drinking was limited. The main intake route for the general population is probably oral (residues on food, contaminated drinking water). In Europe, at the time of the implementation of the presented studies, the use of Gly is mainly limited to pre-seeding applications and as a desiccant [26]. In some EU countries (e.g., Germany) desiccation (= application of herbicide on crops directly before harvest for quicker and more drying) is restricted. The residue levels of Gly in plants in the EU are the highest for pulses and cereals. Only 2–3% of the checked food items contained quantifiably amounts of Gly in 2015 to 2017 [26,38,39,40]. This information is not available for AMPA and it is recommended to also measure AMPA in food items.

If all of the studies are combined, (Supplementary Material Tables S1 and S2), the results from the logistic regression analyses (*p* value set at < 0.2) showed lower urinary Gly concentrations in summer (OR: 0.60 (95%CI 0.35 to 1.03) compared to spring), higher concentrations with pets in the home (OR 1.25 (95%CI 0.90 to 1.74)) and lower concentrations with proximity to agricultural fields (OR 0.57 (95%CI 0.28 to 1.16) for <150 m compared to 150–1000 m) (see Table S1). The latter association was determined by the fact that all of the data in one study (sampling site) were at a certain distance from agricultural fields, while in another study (sampling site) they were all at another distance, although the country was included in the analysis as a covariate. It is thus not trustworthy. The variable proximity to agricultural fields is also a rough indicator as it only contained three distance categories and the location of the house versus the field, taking into account the main wind direction and number of fields in the neighborhood, was not accounted for. For AMPA, there was a negative association with the degree of urbanization (DEGURBA) (OR 0.72 (95%CI 0.42 to 1.20) in rural areas compared to cities) and sampling season (OR 0.52 (95% CI 0.31 to 0.88) for fall compared to spring). In addition, for AMPA, differences in the AMPA concentrations were found regarding the distance to agricultural fields but, as explained above, this is an artefact as the data from different studies were sampled at different distances (categories) from agricultural fields. This was also the case for the DEGURBA. Overall, when the data of the different studies were combined, logistic regression did not show clear and significant associations with the exposure determinants. In addition, no multiple regression with backward selection could be performed on all of the studies combined, seeing that the studied determinants were not available in all of the considered studies.

Other recent studies also reported on the exposure determinants related to Gly exposure in children. In general, Lemke et al. (2021) could not identify specific subgroups with higher internal exposure to Gly or AMPA among the children and adolescents of GerES V [26]. No sex differences were observed in this study. In the study of Stajnko et al. (2020) in Slovenian children and adolescents, higher concentrations were observed in adolescent boys than girls, while no sex difference was seen among children at a lower age (<10 y) [27]. However, Ferreira et al. (2021) found an opposite pattern, with higher concentrations in girls compared to boys in Portugal [24].

Regarding age, the average Gly concentration (µg/L) was reported to increase with age between 2 and 13 yrs. in the study of Ferreira et al. (2021) [24]. Lemke et al. (2021) reported a clear decreasing gradient with increasing age between 3 and 17 yrs. for creatinine-adjusted Gly concentrations, related to higher creatinine concentrations in older children [26]. Stajnko et al. (2020) observed that children rather than adolescents tended to have higher exposure (concentrations adjusted by specific gravity) [27]. When BMI is considered, Stajnko et al. (2020) did not observe higher Gly concentrations with higher BMI [27]. In contrast, a positive association between Gly and BMI was observed by Conrad et al. (2017) in German adults, and deserves attention when further investigating the exposure determinants of Gly, such as food consumption [41]. In our study, BMI was forced into the regression models because of the possible association with creatinine and the association of Gly and AMPA concentrations with BMI in some studies. However, when analyzing the exposure determinants related to food, adjusting for BMI might be a proxy for adjusting for a specific food category (e.g., fast food) which, in this case, will increase the chance that it will not be observed in the regression analysis studying the specific food category but, in general, the associations with food were far from significant.

Higher Gly concentrations were observed in the urine of children living up to 1 km from agricultural areas in Danish children [25] and Portuguese children [24]. However, in the study from Stajnko et al. (2020), no significant associations between urinary Gly or AMPA concentrations and the vicinity of agriculture, vineyards, orchards, gardens, playground/courts, railways or cemeteries and the type of residence area (center/suburbs of a settlement/countryside) were found [27]. Further, in this study of Stajnko, two sampling periods (January–March 2018 and May–June 2018) were selected [27], but no significant difference between the periods was observed.

An increased concentration of Gly in children was noticed in children with higher consumption of home-produced (local) foods in Portugal [24]. Stajnko et al. (2020) did find a higher exposure among the individuals with a higher consumption of nuts and wholegrain rice [27]. No clear association between Gly or AMPA and a vegetarian diet or the consumption of cereals, pulses or vegetables was identified by Lemke et al., 2021 [26].

For educational level, it was found in Portuguese children that when the parents’ educational level increased, the Gly concentrations in the urine of children decreased [24]. Lemke et al. (2021) reported higher volume-based concentrations of Gly in children with medium socioeconomic status (SES), compared to high or low SES [26].

Higher concentrations of Gly were detected in the study of Ferreira et al. (2021) in children’s urine when the parents declared the use of pesticides in the garden versus parents who declared not to use pesticides [24]. In addition, Stajnko et al. (2020) did observe, for one participant, a clear increase in urinary Gly with information on the use near the child’s home [27].

In the literature, no overwhelming evidence of specific exposure determinants was found, as was the case in this study. The currently used questionnaires do not focus on food consumption data 24 h before urinary sampling. The half-life of Gly in the human body is approximately 5 to 10 h [42], and so Gly levels in urine are quite dependent on recent exposures. Therefore, more substance-specific questionnaires are needed. Further, information about vegetarians, organic food consumption and detailed information on the proximity to different types of agricultural fields is needed.

Quality-assured aligned HBM data were collected within HBM4EU. This allowed for the comparison of exposures across the participating countries. For Gly and AMPA, the detection frequencies were relatively low. Dichotomization has the advantage that all of the data were used, but in this way information on actual concentrations was lost.

The main limitations in this study are the relatively low detection frequencies of Gly and AMPA in urine, influencing the exposure- and risk assessment and the analysis of exposure determinants, as well as the low FUE (0.57%) which renders some uncertainty to the reverse dosimetry. The uncertainties in the risk assessment are, e.g., the standardized urinary volume excreted in 24 h and standardized bodyweight and not accounting for similarities in toxicity for AMPA and Gly.

Due to the fact that Gly is ubiquitous, and the uncertainty about the health effects and the (metal) chelating properties [4], further human biomonitoring remains necessary. New biomarkers and matrices for Gly should be explored, as the urinary excretion factor is low (set to 0.57% in this study for the risk assessment). The residue levels of AMPA in food are currently not monitored in the EU monitoring program. The information about AMPA levels in food is thus limited at the moment.

## 4. Conclusions

HBM4EU provided new data (harmonized within the HBM4EU according to chemical and statistical analyses) on the exposure of children to glyphosate and its environmental metabolite, AMPA. These data show that Gly exposure is low but widespread in several EU countries. More HBM data are needed to obtain a full picture across the EU, such as the exposure of specific subpopulations (e.g., living close to agricultural fields; vegetarians) and more harmonized HBM data (with identical LOQ for chemicals with observed concentrations near the LOQ) in other EU countries. By providing these data, the policies protecting human health can be driven more by solid science. Glyphosate has a relatively short half-life. By the measurement of Gly and AMPA in urine and blood, our understanding of the absorption, distribution and elimination of Gly can improve. The comparison of internal HBM data with external exposure, based on food intake information together with Gly- and AMPA-food concentrations, will lead to better models predicting exposure. This should go hand in hand with harmonized questionnaire data on food consumption shortly before the sampling, to obtain a better insight into the exposure determinants. Current analysis shows that, overall, there are limited associations between expected determinants influencing Gly and AMPA urinary levels. Logistic regression did find use of Gly at home and the presence of pets as possible determinants in some of the individual studies. In addition, an improvement in the sensitivity of the analytical methods leading to lower LOQs would enable more detailed analyses of the potential exposure determinants. The finding of sources of AMPA in the environment and the intake routes would help in the risk assessment and management. Glyphosate and AMPA have similar properties and the setting of a group ADI for Gly+AMPA should be considered. There is still a conflict of opinions between IARC and the EU bodies on the genotoxicity as well as the carcinogenicity of Gly, and the outcome of any risk assessment is very much dependent on this categorization. It is not up to the authors in this paper to take a side, and we leave strong conclusions up to the reader. Certainly, the newly proposed ADI of 0.1 mg/kg bw/day makes the margin between real life exposure and safe exposure levels five times smaller than with the previous ADI of 0.5 mg/kg bw/day. A recalculation of the currently found urinary Gly to external exposure and comparison with the new ADI suggests no concern for the children who were investigated in HBM4EU. Further work should focus on the children living in close proximity to the agricultural uses of Gly and the workers involved in the handling of Gly.

## Figures and Tables

**Table 1:** Urinary Gly concentrations in European children (6–11 yrs.) from HBM4EU-aligned studies.

Study	Sampling Year	N	Age Range (yrs.)	Sex (F/M)	Urine Sample	Method	LOD Gly (µg/L)	LOQ Gly (µg/L)	% < LOQ ^f^	Gly (µg/L)				Creatinine-Adjusted Gly (µg/g crt) ^g^				CreatinineMedian (P5 and P95) in mg/dL
										P25	P50	P75	P95	P25	P50	P75	P95	
SLO CRP children (Slovenia) ^a^	2018	149	7–10	(82/67)	Morning (140) and spot (9)	GC/MS-MS		0.1	76	<LOQ	<LOQ	<LOQ	0.18				0.19	102 (42–175)
ORGANIKO (Cyprus) ^b^	2017	166	10–11	(80/86)	Morning	GC/MS-MS	0.03	0.1	55	<LOQ	<LOQ	0.18	1.03			0.15	0.76	108 (53–205)
GerES V-sub ^d^ (Germany) ^a^	2015–2017	300	6–12 ^c^	(150/150)	Morning (296) and spot (4)	GC/MS-MS		0.1	47	<LOQ	0.10 = LOQ	0.17	0.43		0.07	0.15	0.37	110 (49–204)
3XG (Belgium) ^b^	2019–2020	133	6–8	(67/66)	Morning (110) and spot (23)	LC/MS-MS		0.1	40	<LOQ	0.12	0.22	0.41		0.11	0.21	0.58	97 (33–166)
ESTEBAN (France) ^a^	2014–2016	223	6–11	(115/108)	Morning	LC/MS-MS	0.02	0.05	83	<LOQ	<LOQ	<LOQ	0.65				0.84	87 (32–164)
Combined data ^e^		748	6–12	(494/477)								0.15	0.48			0.13	0.51	

^a^: Biomarker data generated before HBM4EU QA/QC program and comparability cannot be guaranteed (see Esteban López et al., 2021) [17]; ^b^: Biomarker data quality assured by HBM4EU QA/QC program; ^c^: GerES V-sub included 19 children who have turned 12 between the first examination and urine collection date. ^d^: GerES V-sub is an unweighted subset of 300 children of the nationally representative GerES V; ^e^: Results of all datasets combined regardless of result QA/QC program; ^f^: Each study was compared to its own LOQ. Data represent the percentage of participants having a value lower than the LOQ; ^g^: Values < LOQ set to LOQ/2.

**Table 2:** Summary of urinary AMPA concentrations in European children (6–11 yrs.) from the HBM4EU-aligned studies.

Study	LOD AMPA (µg/L)	LOQ AMPA (µg/L)	% < LOQ	AMPA (µg/L)				Creatinine-Adjusted AMPA (µg/g crt) ^c^			
				P25	P50	P75	P95	P25	P50	P75	P95
SLO CRP children ^a^		0.1	53	<LOQ	<LOQ	0.14	0.29			0.13	0.26
ORGANIKO ^b^	0.03	0.1	25	=LOQ	0.17	0.28	0.66	0.05	0.15	0.23	0.57
GerES V-sub ^a^		0.1	53	<LOQ	<LOQ	0.21	0.48			0.17	0.40
3XG ^b^		0.2	74	<LOQ	<LOQ	0.20	0.40			0.14	0.37
ESTEBAN ^a^	0.02	0.05	4	0.11	0.18	0.24	0.43	0.12	0.19	0.30	0.59
Combined data						0.22	0.47			0.21	0.45

^a^: Biomarker data generated before HBM4EU QA/QC program but deemed comparable (see Esteban López et al., 2021) [17]; ^b^: Biomarker data quality assured by HBM4EU QA/QC program; ^c^: Values <LOQ set to LOQ/2.

**Table 3:** Urinary Gly and AMPA concentrations in children reported in other European studies.

Study	Country	Sampling Year	Population	Urine Sample	Method (Instrumentation)	Gly, µg/L			AMPA, µg/L		
						LOQ/LOQ	Average	P95	LOD/LOQ	Average	P95
Ferreira et al. 2021 [24]	Portugal	2018–2019	41 children 2–13 y	Spot	ELISA	LOD = 0.6	AM = 1.77				
Knudsen et al. 2017 [25]	Denmark	2011	14 children 6–11 y	Spot	ELISA	LOD = 0.08	AM = 1.96				
Lemke et al. 2021 [26] ^a^	Germany	2015–2017	2144 children and adolescents 3–17 y	First morning	GC/MS-MS	LOQ = 0.1	AM = 0.16 GM = 0.11	0.51	LOQ = 0.1	AM = 0.16 GM = 0.10	0.48
Stajnko et al. 2020 [27] ^b^	Slovenia	2018	246 children and adolescents 7–10 y and 12–15 y	First morning (January–March)	GC/MS-MS	LOQ = 0.1	GM < LOQ	0.19	LOQ = 0.1	GM < LOQ	0.29
			225 children and adolescents 7–10 y and 12–15 y	Fist morning (May–June)	GC/MS-MS	LOQ = 0.1	GM < LOQ	0.19	LOQ = 0.1	GM = 0.1	0.33

LOD: limit of detection; LOQ: limit of quantification; AM: arithmetic mean; GM: geometric mean; P95: 95th percentile. ^a^: results of publication Lemke et al. (2021) [26] include all participants of GerES V (i.e., children + adolescents); ^b^: results of publication Stajnko et al. (2020) [27] include children and adolescents.

**Table 4:** Risk assessment of glyphosate based on HBM data (detection of Gly and AMPA with mass spectrometry).

Ref.	Country	P95 Concentration Glyphosate	PDI	PDI	PDI/ADI
		µg/L	µg/day	µg/kg bw/day	%
SLO CRP children (HBM4EU)	Slovenia	0.18	=0.18 × 0.7/0.57% = 22	=22/30 = 0.74	=0.00074/0.1 = 0.74%
ORGANIKO (HBM4EU)	Cyprus	1.03	126	4.22	4.22%
GerES V-sub (HBM4EU)	Germany	0.43	53	1.76	1.76%
3XG (HBM4EU)	Belgium	0.41	50	1.68	1.68%
ESTEBAN (HBM4EU)	France	0.65	80	2.66	2.66%
Lemke et al. 2021 [26]	Germany	0.51	63	2.09	2.09%
Stajnko et al. 2020 [27]	Slovenia	0.19	23	0.78	0.78%

PDI: predicted daily intake; ADI: acceptable daily intake; P95: 95th percentile. Assumed bodyweight set at 30 kg, urinary volume at 0.7 L/day and F_UE_ at 0.57%. ADI was set at 0.1 mg/kg bw/day.

**Table 5:** Logistic backward multiple regression for glyphosate and AMPA based on individual studies from HBM4EU.

	Study	Variable	OR = Exp(β)	95%CI	*p* Value
Glyphosate	Slovenia SLO_CRP children (HBM4EU)	Intercept	0.15		
		Creatinine	1.01	(1.00;1.02)	0.041
		Matrix	1.08	(0.19;5.96)	0.933
		BMI	0.98	(0.87;1.10)	0.698
		Use of pesticide outdoor (No: 76; Yes: 73)	2.37	(1.07;5.25)	**0.034**
		Model			**0.070**
	Belgium 3XG (HBM4EU)	Intercept	0		
		Creatinine	1.02	(1.01;1.03)	0.001
		Matrix	1.08	(0.38;3.07)	0.890
		BMI	1.03	(0.84;1.26)	0.782
		Age (6–8 y)	2.61	(1.12;6.06)	**0.026**
		Pets in home (No: 80; Yes: 53)	2.73	(1.20;6.21)	**0.017**
		Model			**<0.001**

OR: odds ratio. BMI, creatinine (crt) and matrix (spot urine; first-morning urine) were forced into the model. Only multiple regression models with *p* less than or close to 0.05 shown.

## Data Availability

Data supporting results can be found at www.HBM4EU.eu (accessed on 4 July 2022) and https://www.hbm4eu.eu/what-we-do/european-hbm-platform/eu-hbm-dashboard/ (accessed on 4 July 2022).

## Supplementary Materials

The following supporting information can be downloaded at: https://www.mdpi.com/article/10.3390/toxics10080470/s1, Table S1. Logistic regression for urinary concentrations of glyphosate by some potential exposure determinants, subgroups of the participants stratified for individual characteristics, dietary preferences, exposure relevant behaviour and sociodemographic information. Table S2. Logistic regression for urinary concentrations of AMPA by some potential exposure determinants, subgroups of the participants stratified for individual characteristics, dietary preferences, exposure relevant behaviour and sociodemographic information.

## References

[1] EFSA (2015). Conclusion of the Peer Review of the Pesticide Risk Assessment of the Active Substance Glyphosate. EFSA J..

[2] JMPR Pesticide Residues in Food 2019-Evaluations 2019 Part I-Residues. Proceedings of the EXTRA Joint FAO/WHO Meeting 2019.

[3] Grandcoin A., Piel S., Baurès E. (2017). AminoMethylPhosphonic Acid (AMPA) in Natural Waters: Its Sources, Behavior and Environmental Fate. Water Res..

[4] Jayasumana C., Gunatilake S., Siribaddana S. (2015). Simultaneous Exposure to Multiple Heavy Metals and Glyphosate May Contribute to Sri Lankan Agricultural Nephropathy. BMC Nephrol..

[5] Tarazona J.V., Court-Marques D., Tiramani M., Reich H., Pfeil R., Istace F., Crivellente F. (2017). Glyphosate Toxicity and Carcinogenicity: A Review of the Scientific Basis of the European Union Assessment and Its Differences with IARC. Arch. Toxicol..

[6] De Araújo-Ramos A.T., Passoni M.T., Romano M.A., Romano R.M., Martino-Andrade A.J. (2021). Controversies on Endocrine and Reproductive Effects of Glyphosate and Glyphosate-Based Herbicides: A Mini-Review. Front. Endocrinol..

[7] Silver M.K., Fernandez J., Tang J., McDade A., Sabino J., Rosario Z., Vega C.V., Alshawabkeh A., Cordero J.F., Meeker J.D. (2021). Prenatal Exposure to Glyphosate and Its Environmental Degradate, Aminomethylphosphonic Acid (AMPA), and Preterm Birth: A Nested Case-Control Study in the PROTECT Cohort (Puerto Rico). Environ. Health Perspect..

[8] Parvez S., Gerona R.R., Proctor C., Friesen M., Ashby J.L., Reiter J.L., Lui Z., Winchester P.D. (2018). Glyphosate Exposure in Pregnancy and Shortened Gestational Length: A Prospective Indiana Birth Cohort Study. Environ. Health.

[9] Lesseur C., Pathak K.V., Pirrotte P., Martinez M.N., Ferguson K.K., Barrett E.S., Nguyen R.H.N., Sathyanarayana S., Mandrioli D., Swan S.H. (2021). Urinary Glyphosate Concentration in Pregnant Women in Relation to Length of Gestation. Environ. Res..

[10] Lesseur C., Pirrotte P., Pathak K.V., Manservisi F., Mandrioli D., Belpoggi F., Panzacchi S., Li Q., Barrett E.S., Nguyen R.H.N. (2021). Maternal Urinary Levels of Glyphosate during Pregnancy and Anogenital Distance in Newborns in a US Multicenter Pregnancy Cohort. Environ. Pollut..

[11] Glyphosate: No Change Proposed to Hazard Classification. https://echa.europa.eu/-/glyphosate-no-change-proposed-to-hazard-classification#:~:text=ECHA’sCommitteeforRiskAssessment,acarcinogenisnotjustified.

[12] Makris K.C., Efthymiou N., Konstantinou C., Anastasi E., Schoeters G., Kolossa-Gehring M., Katsonouri A. (2022). Oxidative Stress of Glyphosate, AMPA and Metabolites of Pyrethroids and Chlorpyrifos Pesticides among Primary School Children in Cyprus. Environ. Res..

[13] Davoren M.J., Schiestl R.H. (2018). Glyphosate-Based Herbicides and Cancer Risk: A Post-IARC Decision Review of Potential Mechanisms, Policy and Avenues of Research. Carcinogenesis.

[14] Ganzleben C., Antignac J.-P., Barouki R., Castaño A., Fiddicke U., Klánová J., Lebret E., Olea N., Sarigiannis D., Schoeters G.R. (2017). Human Biomonitoring as a Tool to Support Chemicals Regulation in the European Union. Int. J. Hyg. Environ. Health.

[15] Gilles L., Govarts E., Rambaud L., Vogel N., Castaño A., Esteban López M., Rodriguez Martin L., Koppen G., Remy S., Vrijheid M. (2021). HBM4EU Combines and Harmonises Human Biomonitoring Data across the EU, Building on Existing Capacity—The HBM4EU Survey. Int. J. Hyg. Environ. Health.

[16] Gilles L., Govarts E., Rodriguez Martin L., Andersson A.-M., Appenzeller B.M.R., Barbone F., Castaño A., Coertjens D., Den Hond E., Dzhedzheia V. (2022). Harmonisation of Human Biomonitoring Studies in Europe: Characteristics of the HBM4EU Aligned Studies Participants. Int. J. Environ. Res. Public Health.

[17] Esteban López M., Göen T., Mol H., Nübler S., Haji-Abbas-Zarrabi K., Koch H.M., Kasper-Sonnenberg M., Dvorakova D., Hajslova J., Antignac J.P. (2021). The European Human Biomonitoring Platform—Design and Implementation of a Laboratory Quality Assurance/Quality Control (QA/QC) Programme for Selected Priority Chemicals. Int. J. Hyg. Environ. Health.

[18] González-Alzaga B., Hernández A.F., Kim Pack L., Iavicoli I., Tolonen H., Santonen T., Vinceti M., Filippini T., Moshammer H., Probst-Hensch N. (2022). The Questionnaire Design Process in the European Human Biomonitoring Initiative (HBM4EU). Environ. Int..

[19] Sarigiannis D., Horvat M. (2018). Report on the Optimal Methodology for Exposure Reconstruction from HBM Data Deliverable Report D 12.2 HBM4EU. https://www.hbm4eu.eu/work-packages/deliverable-12-2-report-on-the-optimal-methodology-for-exposure-reconstruction-from-hbm-data/.

[20] EFSA (2021). EFSA–Renewal Assessment Report–Open for Consultation.

[21] Zoller O., Rhyn P., Zarn J.A., Dudler V. (2020). Urine Glyphosate Level as a Quantitative Biomarker of Oral Exposure. Int. J. Hyg. Environ. Health.

[22] Connolly A., Coggins M.A., Koch H.M. (2020). Human Biomonitoring of Glyphosate Exposures: State-of-the-Art and Future Research Challenges. Toxics.

[23] Arpilleda J.C. (2010). Evidence-Based Management of Pediatric Genitourinary Tract Injuries in the ED (Trauma CME).

[24] Ferreira C., Duarte S.C., Costa E., Pereira A.M.P.T., Silva L.J.G., Almeida A., Lino C., Pena A. (2021). Urine Biomonitoring of Glyphosate in Children: Exposure and Risk Assessment. Environ. Res..

[25] Knudsen L.E., Hansen P.W., Mizrak S., Hansen H.K., Mørck T.A., Nielsen F., Siersma V., Mathiesen L. (2017). Biomonitoring of Danish School Children and Mothers Including Biomarkers of PBDE and Glyphosate. Rev. Environ. Health.

[26] Lemke N., Murawski A., Schmied-Tobies M., Rucic E., Hoppe H., Conrad A., Kolossa-Gehring M. (2021). Glyphosate and Aminomethylphosphonic Acid (AMPA) in Urine of Children and Adolescents in Germany—Human Biomonitoring Results of the German Environmental Survey 2014–2017 (GerES V). Environ. Int..

[27] Stajnko A., Snoj Tratnik J., Kosjek T., Mazej D., Jagodic M., Eržen I., Horvat M. (2020). Seasonal Glyphosate and AMPA Levels in Urine of Children and Adolescents Living in Rural Regions of Northeastern Slovenia. Environ. Int..

[28] Hines E.P., Mendola P., von Ehrenstein O.S., Ye X., Calafat A.M., Fenton S.E. (2015). Concentrations of Environmental Phenols and Parabens in Milk, Urine and Serum of Lactating North Carolina Women. Reprod. Toxicol..

[29] Gillezeau C., Lieberman-Cribbin W., Taioli E. (2020). Update on Human Exposure to Glyphosate, with a Complete Review of Exposure in Children. Environ. Health.

[30] Trasande L., Aldana S.I., Trachtman H., Kannan K., Morrison D., Christakis D.A., Whitlock K., Messito M.J., Gross R.S., Karthikraj R. (2020). Glyphosate Exposures and Kidney Injury Biomarkers in Infants and Young Children. Environ. Pollut..

[31] Van Bruggen A.H.C., He M.M., Shin K., Mai V., Jeong K.C., Finckh M.R., Morris J.G. (2018). Environmental and Health Effects of the Herbicide Glyphosate. Sci. Total Environ..

[32] Niemann L., Sieke C., Pfeil R., Solecki R. (2015). A Critical Review of Glyphosate Findings in Human Urine Samples and Comparison with the Exposure of Operators and Consumers. J. Verbrauch. Leb..

[33] Brewster D.W., Warren J., Hopkjns W.E. (1991). Metabolism of Glyphosate in Sprague-Dawley Rats: Tissue Distribution, Identification, and Quantitation of Glyphosate-Derived Materials Following a Single Oral Dose. Toxicol. Sci..

[34] Studnik H., Liebsch S., Forlani G., Wieczorek D., Kafarski P., Lipok J. (2015). Amino Polyphosphonates—Chemical Features and Practical Uses, Environmental Durability and Biodegradation. New Biotechnol..

[35] Faniband M.H., Norén E., Littorin M., Lindh C.H. (2021). Human Experimental Exposure to Glyphosate and Biomonitoring of Young Swedish Adults. Int. J. Hyg. Environ. Health.

[36] JMPR Pesticides residues in food 2011. Proceedings of the Joint FAO/WHO Meeting on Pesticide Residues, Glyphosate and Metabolites.

[37] Nishioka M.G., Lewis R.G., Brinkman M.C., Burkholder H.M., Hines C.E., Menkedick J.R. (2001). Distribution of 2,4-D in Air and on Surfaces inside Residences after Lawn Applications: Comparing Exposure Estimates from Various Media for Young Children. Environ. Health Perspect..

[38] EFSA (2017). The 2015 European Union Report on Pesticide Residues in Food. EFSA J..

[39] EFSA (2018). The 2016 European Union Report on Pesticide Residues in Food. EFSA J..

[40] EFSA (2019). The 2017 European Union Report on Pesticide Residues in Food. EFSA J..

[41] Conrad A., Schröter-Kermani C., Hoppe H.W., Rüther M., Pieper S., Kolossa-Gehring M. (2017). Glyphosate in German Adults—Time Trend (2001 to 2015) of Human Exposure to a Widely Used Herbicide. Int. J. Hyg. Environ. Health.

[42] Connolly A., Jones K., Basinas I., Galea K.S., Kenny L., McGowan P., Coggins M.A. (2019). Exploring the Half-Life of Glyphosate in Human Urine Samples. Int. J. Hyg. Environ. Health.

