# Supplementary material for: Glyphosate and AMPA in Human Urine of HBM4EU Aligned Studies: Part A Children
Source: Toxics. 2022 Aug 12;10(8):470. doi: 10.3390/toxics10080470 (PMC9415901; doi:10.3390/toxics10080470)
Supplement: Supplementary file 1 [file toxics-10-00470-s001.zip › toxics-1825547-supplementary.pdf]

Supplementary Materials

Table S1. Logistic regression for urinary concentrations of glyphosate by some potential exposure determinants, subgroups of the participants stratified for individual characteristics, dietary preferences, exposure relevant behaviour and sociodemographic information.

Determinant		SLO_CRP children			ORGANIKO			GerES V-sub			3XG			ESTEBAN			Combined data*		
		N	Beta	p value	N	Beta	p value	N	Beta	p value	N	Beta	p value	N	Beta	p value	N	Beta	p value
Sex	Female	82	0		76	0		150	0		67	0		115	0		490	0	
	Male	67	0.03	0.939	82	-0.19	0.576	150	-0.24	0.324	66	0.18	0.641	108	0.101	0.779	473	-0.08	0.541
Age in years		149	-0.09	0.609	158	-0.02	0.951	300	-0.07	0.302	133	1.03	0.015	223	0.178	0.165	963	-0.01	0.854
Nuts 1		all same category			All same category			300		0.469	All same category			223		0.621	963		<0.001
Nuts 2		all same category			All same category						All same category			223		0.922	300		<0.001
Nuts 3		all same category			All same category									223		1	433		1
DEGURBA	Densely populated area (cities)							65	0					52	0		117	0	
	Intermediate density area (towns or suburbs)							132	-0.07	0.824	All same category			67	0.652	0.173	332	0.122	0.643
	Thinly populated area (rural area)	all same category						103	-2.97	0.374				104	-0.239	0.626	356	-0.296	0.271
Sampling season	Spring							109	0		21	0		55	0		185	0	
	Summer							37	-0.52	0.191	41	-0.69	0.251	42	-0.340	0.576	120	-0.512	0.063
	Fall							70	-0.007	0.984	25	0.25	0.715	51	0.378	0.454	146	0.096	0.697
	Winter	all same category			All same category			84	-0.05	0.883	46	-0.43	0.467	75	0.102	0.832	512	-0.139	0.546
Use of pesticide outdoor	No	76	0		124	0					126	0		97	0		423	0	
	Yes	73	0.86	0.034	33	-0.21	0.614				5	0.6	0.534	80	-0.03	0.950	191	0.256	0.260
Frequency consumption of eggs	0 = Never	3	0		6	0		18	0		3	0		7	0		37	0	
	Rarely: <1 time / month	1	-20.17	1	1	-20.1	1				50	-0.71	0.587	10	-19.41	0.999	62	-0.304	0.556

Determinant		SLO_CRP children			ORGANIKO			GerES V-sub			3XG			ESTEBAN			Combined data*		
		**N**	**Beta**	**p value**	**N**	**Beta**	**p value**	**N**	**Beta**	**p value**	**N**	**Beta**	**p value**	**N**	**Beta**	**p value**	**N**	**Beta**	**p value**
	Sometimes: <= 1 time / week but >= 1 time/month	52	-0.96	0.458	18	1.15	0.354	250	-0.02	0.963				159	0.23	0.836	479	0.054	0.889
	Often: 2-3 times / week	67	-0.73	0.571	71	1.32	0.25	20	-0.22	0.744	79	-0.48	0.714	33	0.67	0.559	270	0.156	0.702
	Very Often: 4-6 times / week	20	-0.2	0.879	26	1.45	0.216	3	0.32	0.807				2	-19.46	0.999	51	0.485	0.328
	Everyday: >= 7 times / week	6	-20.7	0.999	4	0.97	0.538	5	-0.82	0.459							15	-0.747	0.315
Frequency consumption of fruit	0 = Never	2	0		1	0		2	0		1	0		2	0		8	0	
	Rarely: <1 time / month	1	42.3	0.999										2	-21.27	0.999	3	1.46	0.336
	Sometimes: <= 1 time / week but >= 1 time/month	6	-0.23	1	6	-20.32	1	35	21.63	0.999				10	-0.88	0.579	57	0.66	0.471
	Often: 2-3 times / week	21	20.41	0.999	27	-20.22	1	38	21.36	0.999	3	21.67	1	38	-3.67	0.036	127	0.53	0.553
	Very Often: 4-6 times / week	38	19.76	0.999	18	-21.75	1	33	22.13	0.999	16	21.19	1	60	-1.29	0.377	165	0.70	0.432
	Everyday: >= 7 times / week	81	20.29	0.999	74	-20.98	1	189	21.56	0.999	113	21.02	1	98	-1.52	0.292	555	0.65	0.454
Frequency consumption of vegetables	0 = Never				9	0		7	0					2	0		18	0	
	Rarely: <1 time / month													2	-21.27	0.999	2	-19.76	0.999
	Sometimes: <= 1 time / week but >= 1 time/month	1	0		117	-0.24	0.744	84	0.05	0.949				10	-0.88	0.579	212	-0.24	0.653

Determinant		SLO_CRP children			ORGANIKO			GerES V-sub			3XG			ESTEBAN			Combined data*		
		N	Beta	p value	N	Beta	p value	N	Beta	p value	N	Beta	p value	N	Beta	p value	N	Beta	p value
	Often: 2-3 times / week	8	20.54	1				69	0.09	0.921	7	0		38	-3.67	0.036	122	-0.43	0.450
	Very Often: 4-6 times / week	14	20.63	1				44	-0.04	0.964	27	-0.65	0.526	60	-1.29	0.377	145	-0.13	0.824
	Everyday: >= 7 times / week	126	20.25	1				93	0.11	0.895	99	-1.04	0.282	98	-1.52	0.292	416	-0.27	0.626
Frequency consumption of cereals	0 = Never							1	0					3	0		4	0	
	Rarely: <1 time / month																160	0.42	0.729
	Sometimes: <= 1 time / week but >= 1 time/month	3	0					135	-20.95	1				22	19.74	0.999	214	0.13	0.914
	Often: 2-3 times / week	30	20	0.999				75	-21.31	1	1	0		108	19.57	0.999	162	0.39	0.747
	Very Often: 4-6 times / week	63	19.84	0.999				28	-20.73	1	6	21.31	1	65	19.79	0.999	251	0.33	0.788
	Everyday: >= 7 times / week	53	19.96	0.999				59	-21.22	1	126	21.43	1	13	20.08	0.999			
Frequency consumption of local food	0 = Never	2	0								48	0					50	0	
	Rarely: <1 time / month										25	0.02	0.969				25	0.03	0.949
	Sometimes: <= 1 time / week but >= 1 time/month										29	0.41	0.438				29	0.36	0.478
	Often: 2-3 times / week	9	19.27	0.999							15	0.32	0.634				24	0.01	0.981
	Very Often: 4-6 times / week	14	20.25	0.999													14	-0.05	0.948

Determinant		SLO_CRP children			ORGANIKO			GerES V-sub			3XG			ESTEBAN			Combined data*		
		**N**	**Beta**	**p value**	**N**	**Beta**	**p value**	**N**	**Beta**	**p value**	**N**	**Beta**	**p value**	**N**	**Beta**	**p value**	**N**	**Beta**	**p value**
	Everyday : >= 7 times / week	124	20.13	0.999							16	-0.69	0.277				140	-0.22	0.669
Frequency consumption of organic food	0 = Never																		
	Rarely: <1 time / month													65	0		65	0	
	Sometimes: <= 1 time / week but >= 1 time/month													36	-0.13	0.803	36	-0.13	0.803
	Often: 2-3 times / week													27	-2.03	0.059	27	-2.03	0.059
	Very Often: 4-6 times / week													22	-0.61	0.391	22	-0.61	0.391
	Everyday : >= 7 times / week													61	-0.51	0.295	61	-0.51	0.295
Vegetarian	No	all same category						283	0		80	0		All same category for for available data (those who answered question)			530	0	
	Yes							13	0.65	0.303	2	-0.41	0.785				15	0.524	0.362
Type of drinking water most consumed	Bottled water	25	0											51	0		76	0	
	Tap water	120	-0.88	0.073										132	-0.272	0.562	252	-0.16	0.619
	Ground water	4	-20.68	0.999										8	-0.112	0.922	12	-1.10	0.310
	Other													24	0.506	0.438	24	0.12	0.841
Tap water source at home	Public	144	0	1				all same category			63	0					499	0	
	Private well	4	-19.95	0.999													4	-19.76	0.999

Determinant		SLO_CRP children			ORGANIKO			GerES V-sub			3XG			ESTEBAN			Combined data*		
		N	Beta	p value	N	Beta	p value	N	Beta	p value	N	Beta	p value	N	Beta	p value	N	Beta	p value
	Both public and private well	1	-19.79	1							57	-0.49	0.212				58	-0.52	0.178
Frequency of consumption of tea and coffee	0 = Never	138	0					65	0					122	0		325	0	
	Rarely: <1 time / month	2	22.49	0.999										22	-1.02	0.191	24	-0.01	0.993
	Sometimes: <= 1 time / week but >= 1 time/month	4	1.26	0.222				133	0.08	0.799				42	-0.70	0.182	179	-0.08	0.728
	Often: 2-3 times / week	3	-19.9	0.999				31	-0.32	0.498				13	-0.42	0.600	47	-0.46	0.234
	Very Often: 4-6 times / week	1	-20.11	1				10	0.01	0.987				2	-19.93	0.999	13	-0.34	0.592
	Everyday : >= 7 times / week	1	-20.19	1				59	0.16	0.676				10	-0.90	0.404	70	-0.09	0.767
Are there pets in home	No	115	0					172	0		80	0		79	0		446	0	
	Yes	34	0.17	0.709				128	-0.12	0.628	53	1.06	0.01	136	0.17	0.660	351	0.235	0.164
Proximity to agricultural fields	>1000 m																		
	Between 150 and 1000 m	all same category															149	0	
	< 150 m							All same category for for available data						All same category for for available data (those who answered question)			166	-0.57	0.121
Income by household**	Low	12	0								1	0		10	0		23	0	
	Medium	72	-0.49	0.486							14	-19.18	1	78	0.20	0.811	164	-0.28	0.583
	High	48	-0.46	0.52	All same category						116	-19.05	1	121	-0.65	0.439	285	-0.59	0.251

Determinant		SLO_CRP children			ORGANIKO			GerES V-sub			3XG			ESTEBAN			Combined data*		
		N	Beta	p value	N	Beta	p value	N	Beta	p value	N	Beta	p value	N	Beta	p value	N	Beta	p value
	Don't know or don't want to share										1	2.55	1	14	0.48	0.626	15	0.36	0.636
Highest education household	ISCED 0-2	10	0					15	0		1	0		10	0		36	0	
	ISCED 3-4	39	0.92	0.422				116	0.58	0.314	19	-19.94	1	76	-0.17	0.839	250	0.46	0.282
	ISCED≥5	100	1.19	0.276				169	0.43	0.444	111	-20.72	1	136	-0.19	0.821	673	0.32	0.434

p values mentioned are pairwise p values except for age which was not categorical but continuous

Covariates forced into the model were creatinine, BMI, matrix (morning & spot urine); for combined studies also country was forced into the model.

OR= exp (Beta)

*: LOQ set at 0.1 µg/L

**: Country specific

Note that if a trend was observed in the data and the distribution of participants was unequal over different categories or the number of participants was small for certain categories, a combination of categories was taken. Here results are displayed as observed in the original questionnaire.

Not all studied exposure determinants are listed in the table above; Most relevant were chosen.

Table S2. Logistic regression for urinary concentrations of AMPA by some potential exposure determinants, subgroups of the participants stratified for individual characteristics, dietary preferences, exposure relevant behaviour and sociodemographic information.

Determinant		SLO_CRP children			ORGANIKO			GerES V-sub			3XG			ESTEBAN			Combined data*		
		N	Beta	p value	N	Beta	p value	N	Beta	p value	N	Beta	p value	N	Beta	p value	N	Beta	p value
Sex	Female	82	0		76	0		150			67	0		115	0		423	0	
	Male	67	0.55	0.118	82	-0.32	0.416	150	-0.19	0.436	66	-0.17	0.692	108	-0.09	0.885	407	-0.12	0.441
Age in years		149	-0.05	0.777	158	0.03	0.934	300	-0.06	0.423	133	0.67	0.181	223	-0.01	0.994	830	-0.05	0.362
Nuts 1		all same category			All same category			300		0.672	All same category			223		0.996	830		<0.001
Nuts 2		all same category			All same category						All same category			223		1	277		<0.001
Nuts 3		all same category			All same category									223		1	303		0.754
DEGURBA	Densely populated area (cities)							65	0					52	0		117	0	
	Intermediate density area (towns or suburbs)							132	0.26	0.408	All same category			67	-0.41	0.640	199	0.06	0.81
	Thinly populated area (rural area)	all same category						103	-0.34	0.304				104	-0.07	0.939	356	-0.33	0.201
Sampling season	Spring							109	0		21	0		55	0		164	0	
	Summer							37	-0.3	0.932	41	-0.58	0.415	42	-17.99	0.997	79	-0.4	0.199
	Fall							70	-0.66	0.047	25	0.64	0.373	51	-18.34	0.997	121	-0.65	0.015
	Winter	all same category			All same category			84	0.11	0.708	46	0.04	0.947	75	-18.39	0.997	466	-0.15	0.555
Use of pesticide outdoor	No	76	0		124	0					126	0		97	0		297	0	
	Yes	73	0.344	0.322	33	-0.57	0.2				5	-20.66	0.999	80	-0.07	0.930	186	0.02	0.911
Frequency consumption of eggs	0 = Never	3	0		6	0		18	0		3	0		7	0		34	0	
	Rarely: <1 time / month	1	-19.9	1	1	20.57	1				50	20.02	0.999	10	19.70	0.999	12	2.17	0.065
	Sometimes: <= 1 time / week but >= 1 time/month	52	0.14	0.915	18	0.85	0.419	250	0.5	0.344				159	1.11	0.340	479	0.42	0.27

Determinant		SLO_CRP children			ORGANIKO			GerES V-sub			3XG			ESTEBAN			Combined data*		
		N	Beta	p value	N	Beta	p value	N	Beta	p value	N	Beta	p value	N	Beta	p value	N	Beta	p value
	Often: 2-3 times / week	67	-0.1	0.936	71	0.81	0.368	20	-0.13	0.853	79	19.63	0.999	33	1.73	0.248	191	0.31	0.449
	Very Often: 4-6 times / week	20	0.22	0.868	26	1.09	0.27	3	-0.31	0.817				2	19.32	0.999	51	0.49	0.324
	Everyday: >= 7 times / week	6	0.38	0.798	4	0.49	0.741	5	-0.14	0.903							15	0.25	0.714
Frequency consumption of fruit	0 = Never	2	0		1	0		2	0		1	0		2	0		7	0	
	Rarely: <1 time / month	1	42.23	0.999										2	20.99	0.999	3	21.99	0.999
	Sometimes: <= 1 time / week but >= 1 time/month	6	21.66	0.999	6	-21.08	1	35	-0.21	0.882				10	2.52	0.168	57	1.07	0.252
	Often: 2-3 times / week	21	21.81	0.999	27	-19.35	1	38	-0.51	0.729	6	0.36	1	38	3.80	0.033	124	1.45	0.108
	Very Often: 4-6 times / week	38	21.13	0.999	18	-19.26	1	33	-0.02	0.991	16	18.74	1	60	3.79	0.021	149	1.61	0.074
	Everyday: >= 7 times / week	81	21.08	0.999	74	-19.37	1	189	0.09	0.946	113	19.62	1	98	3.16	0.037	442	1.45	0.103
Frequency consumption of vegetables	0 = Never				9	0		7	0					2	0		18	0	
	Rarely: <1 time / month													2	20.99	0.999	2	20.46	0.999
	Sometimes: <= 1 time / week but >= 1 time/month	1	0		117	-0.63	0.479	84	0.36	0.674				10	2.52	0.168	212	0.27	0.62
	Often: 2-3 times / week	8	-20.97	1				69	0.59	0.499	7	0		38	3.80	0.033	115	0.6	0.303
	Very Often: 4-6 times / week	14	-21.35	1				44	0.07	0.932	27	20.16	0.999	60	3.79	0.021	118	0.44	0.453

Determinant		SLO_CRP children			ORGANIKO			GerES V-sub			3XG			ESTEBAN			Combined data*		
		N	Beta	p value	N	Beta	p value	N	Beta	p value	N	Beta	p value	N	Beta	p value	N	Beta	p value
	Everyday: >= 7 times / week	126	-20.87	1				93	0.46	0.597	99	19.3	0.999	98	3.16	0.037	317	0.4	0.477
Frequency consumption of cereals	0 = Never							1	0					3	0		4	0	
	Rarely: <1 time / month																		
	Sometimes: <= 1 time / week but >= 1 time/month	3	0					135	-21.35	1				22	0.30	1	160	-20.14	0.999
	Often: 2-3 times / week	30	-1.35	0.302				75	-21.4	1	1	0		108	-17.67	0.999	213	-20.22	0.999
	Very Often: 4-6 times / week	63	-1.31	0.303				28	-21.18	1	6	20.03	1	65	-18.55	0.999	156	-20.44	0.999
	Everyday: >= 7 times / week	53	-0.85	0.504				59	-21.27	1	126	19.97	1	13	0.52	1	125	-20.07	0.999
Frequency consumption of local food	0 = Never	2	0								48	0					2	0	
	Rarely: <1 time / month										25	-0.09	0.884						
	Sometimes: <= 1 time / week but >= 1 time/month										29	0.32	0.582						
	Often: 2-3 times / week	9	21.88	0.999							15	0.35	0.632				9	21.88	0.999
	Very Often: 4-6 times / week	14	21.92	0.999													14	21.99	0.999
	Everyday: >= 7 times / week	124	21.22	0.999							16	-0.41	0.602				124	21.22	0.999
Frequency consumption of organic food	0 = Never																		

Determinant		SLO_CRP children			ORGANIKO			GerES V-sub			3XG			ESTEBAN			Combined data*		
		N	Beta	p value	N	Beta	p value	N	Beta	p value	N	Beta	p value	N	Beta	p value	N	Beta	p value
	Rarely: <1 time / month													65	0		Only data ESTEBAN available		
	Sometimes: <= 1 time / week but >= 1 time/month													36	18.14	0.998			
	Often: 2-3 times / week													27	0.39	0.744			
	Very Often: 4-6 times / week													22	0.36	0.764			
	Everyday: >= 7 times / week													61	-0.42	0.579			
Vegetarian	No	all same category						283	0		80	0		All same category for for available data (those who answered question)			450	0	
	Yes							13	0.62	0.305	2	-19.99	0.999				13	0.65	0.282
Type of drinking water most consumed	Bottled water	25	0											51	0		76	0	
	Tap water	120	-0.18	0.703										132	-0.93	0.393	252	-0.27	0.377
	Ground water	4	1.38	0.264										8	-3.11	0.021	12	-0.55	0.43
	Other													24	-0.94	0.518	24	0.33	0.636
Tap water source at home	Public	144	0					all same categorie			63	0					436	0	
	Private well	4	0.52	0.627													4	0.47	0.649
	Both public and private well	1	22.04	1							57	-0.66	0.159				1	21.98	1
Frequency of consumption of tea and coffee	0 = Never	138	0					65	0					122	0		325	0	
	Rarely: <1 time / month	2	0.21	0.888										22	-1.57	0.060	24	-0.59	0.212

Determinant		SLO_CRP children			ORGANIKO			GerES V-sub			3XG			ESTEBAN			Combined data*		
		N	Beta	p value	N	Beta	p value	N	Beta	p value	N	Beta	p value	N	Beta	p value	N	Beta	p value
	Sometimes: <= 1 time / week but >= 1 time/month	4	1.36	0.253				133	0.01	0.993				42	-17.80	0.998	179	0.15	0.539
	Often: 2-3 times / week	3	-0.36	0.79				31	-0.97	0.053				13	-1.80	0.060	47	-0.54	0.139
	Very Often: 4-6 times / week	1	-21.19	1				10	-1.34	0.114				2	17.91	0.999	13	-1.87	0.021
	Everyday: >= 7 times / week	1	-21.01	1				59	-0.2	0.954				10	-0.91	0.442	70	0.05	0.868
Are there pets in home	No	115	0					172	0		80	0		79	0		366	0	
	Yes	34	0.007	0.986				128	-0.5	0.825	53	0.18	0.682	136	0.01	0.999	298	0.1	0.573
Proximity to agricultural fields	>1000 m																		
	Between 150 and 1000 m	all same category						All same category for available data									149	0	
	< 150 m													All same category for for available data (those who answered			166	1.55	<0.001
Income by household**	Low	12	0								1	0		10	0				
	Medium	72	0.34	0.62							14	17.69	1	78	0.26	0.823	22	0	
	High	48	0.46	0.506	All same category						116	19.25	1	121	1.16	0.326	150	0.25	0.619
	Don't know or don't want to share										1	-0.71	1	14	18.90	0.999	169	0.38	0.45
Highest education household	ISCED 0-2	10	0					15	0		1	0		10	0		14	0.9	0.331
	ISCED 3-4	39	0.4	0.6				116	0.18	0.754	19	20.63	1	76	1.42	0.256	35	0	
	ISCED≥5	100	0.19	0.789				169	0.19	0.731	111	20.33	1	136	1.34	0.253	231	0.11	0.783

p values mentioned are pairwise p values except for age which was not categorical but continuous
Covariates forced into the model were creatinine, BMI, matrix (morning & spot urine)
OR= exp (Beta)
*: LOQ was set at 0.1 µg/L. Study 3XG was not taken into account here seeing the LOQ in that study was already 0.2 µg/L.
**: Country specific
Note that if a trend was observed in the data and the distribution of participants was unequal over different categories or the number of participants was small for certain categories, a combination of categories was taken. Here results are displayed as observed in the original questionnaire.
Not all studied exposure determinants are listed in the table above; Most relevant were chosen.
